# Hemoglobin-to-red blood cell distribution width ratio as a protective factor against coronary artery disease: a cross-sectional analysis of NHANES (2011-2018)

**DOI:** 10.3389/fphar.2025.1534479

**Published:** 2025-01-29

**Authors:** Xin-Da Wang, Chaoya Li, Jia Hu, Fen Cao, Li Zhu, Yongzhi Zhu, Zhongzheng Wen, Jun Liu

**Affiliations:** ^1^ Department of Cardiology, Chenzhou First People’s Hospital, Chenzhou, China; ^2^ Hunan University of Medicine General Hospital, Huaihua, China; ^3^ Department of Cardiology, Chengdu First People’s Hospital, Chengdu, China

**Keywords:** coronary artery disease, hemoglobin-to-red blood cell distribution width ratio, inflammation, NHANES, cross-sectional study

## Abstract

**Background:**

Coronary artery disease (CAD) is the leading cause of death worldwide, and inflammation is a significant factor in its development. While the hemoglobin-to-red blood cell distribution width ratio (HRR), an indicator of inflammation, has been linked to various diseases, its association with CAD is not well established.

**Methods:**

We conducted an analysis using data from the National Health and Nutrition Examination Survey (NHANES) spanning from 2011 to 2018. After excluding participants due to age, missing data, and potential confounding factors, 6,881 individuals were included in our study. CAD was identified through self-reported questionnaires, and HRR was determined from laboratory measurements. We controlled for factors such as hypertension, waist circumference, systolic blood pressure, fasting plasma glucose, and others in our logistic regression analysis to explore the relationship between HRR and CAD.

**Results:**

We found that higher HRR levels were associated with a lower risk of CAD. In our fully adjusted model, the odds ratios for CAD for the second, third, and fourth quartiles of HRR were 0.38, 0.42, and 0.51, respectively, compared to the first quartile (P < 0.001). An increase in HRR by one unit was associated with a 49% decrease in the likelihood of CAD. Furthermore, linear regression models indicated a 74% reduction in CAD risk for each one-unit increase in HRR (P = 0.0002). There was a notable threshold at HRR 1.02; beyond this point, each unit increase in HRR was associated with a 91% decrease in CAD odds. This suggests that for individuals with an HRR above 1.02, strategies to increase body water content and reduce blood viscosity could potentially lower their risk of developing CAD.

**Conclusion:**

Our study revealed an inverse linear relationship between HRR and CAD risk, indicating that HRR may serve as a protective factor against CAD.

## Introduction

Coronary artery disease (CAD) is one of the common diseases in cardiovascular medicine, including but not limited to coronary atherosclerosis, angina, myocardial ischemia, myocardial infarction, and coronary artery spasm (Crook et al., 2023; Chen et al., 2024; Kalyani et al., 2014). According to data from the World Health Organization, 7.4 million people worldwide die from CAD (Szilveszter et al., 2022; Manolis et al., 2024). The incidence of CAD is increasing year by year, and it is also a significant factor in global disability and mortality rates, having a major impact on individual health and socio-economics (Tada et al., 2024; Tsukihashi et al., 2021; Omori et al., 2019). The causes of CAD are diverse, including hypertension (Honigberg et al., 2019; Wang et al., 2023), diabetes (Mäenpää et al., 2023; Lin et al., 2024; Luo et al., 2019), hyperlipidemia (Li et al., 2023; Andersen, 1992), obesity (Yang et al., 2023; Jahangir et al., 2014; Pedersen et al., 2019), inflammation (Attiq et al., 2024; Zhao Z. et al., 2024; Kinoshita et al., 2023), and lifestyle factors (Cho et al., 2023; Fu et al., 2023; Said et al., 2019). Among these, inflammation is an important etiology, and in-depth research on it can help improve outcomes for CAD patients.

The hemoglobin-to-red blood cell distribution width ratio (HRR) is recognized as an inflammatory marker associated with the incidence and adverse events of various diseases (Chen et al., 2023; Zhao W. X. et al., 2024; Del Rosso et al., 2022), such as diabetes (Liu et al., 2016), atrial fibrillation (Ralevic et al., 2016; Cortigiani et al., 2020), and even non-small cell lung cancer (Marzio et al., 2022; Meng et al., 2022). HRR is primarily composed of two hematological indicators: hemoglobin (HB) and red blood cell distribution width (RDW) (Su et al., 2021). HB mainly reflects the degree of anemia, while RDW indicates the heterogeneity in red blood cell volume and is indirectly used for diagnosing anemia (Jang et al., 2023). Previous studies have shown that HRR undergoes dynamic changes in response to disease stimuli, and its level variations can serve as a sensitive measure of inflammation (Yılmaz et al., 2021; Eyiol and Ertekin, 2024). Although HRR, as an inflammatory marker, is related to numerous diseases, the clinical relationship between HRR and CAD remains unclear. The hemoglobin-to-red blood cell distribution width ratio (HRR) is calculated using routine blood parameters, and it has been shown to correlate with systemic inflammation. In the context of CAD, inflammation plays a critical role in plaque formation and destabilization, which can lead to acute cardiovascular events. By examining HRR in relation to CAD, we aim to explore its potential as an accessible and cost-effective biomarker for risk stratification in CAD patients. This study attempts to explore the association between HRR and CAD from the perspective of inflammation.

Given that CAD may be associated with inflammation, there might be a unique link between HRR and CAD. However, the relationship between HRR and CAD is not clear in current research. Therefore, this study investigates whether HRR, an emerging inflammatory marker, is independently associated with CAD prevalence, addressing a significant gap in existing literature.

## Materials and methods

### Study population and study design

**FIGURE 1:**
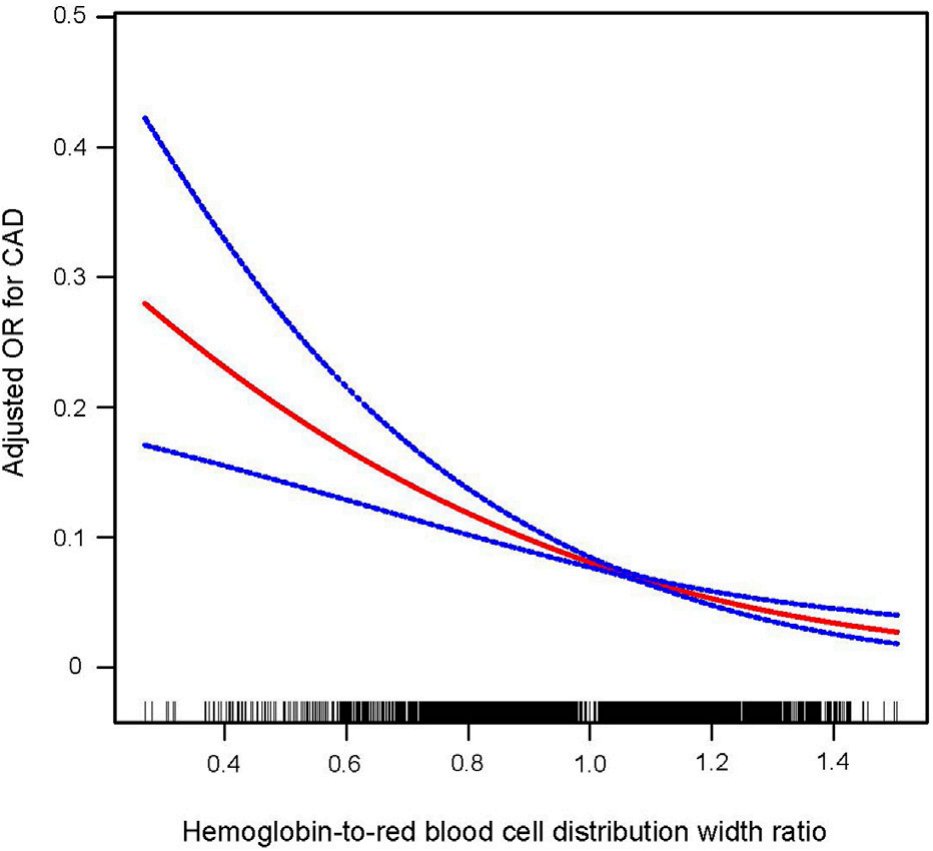
Research flowchart.

NHANES is a cross-sectional, nationally representative survey conducted in the United States. It encompasses data collection through basic information, anthropometric measurements, blood samples, and self-reported questionnaires. Additionally, informed consent is obtained from each participant at the outset of NHANES, thereby eliminating the need for further ethical approval for this study. Figure 1 provides a detailed depiction of the participant selection process for this study (Figure 1). Initially, we extracted demographic, laboratory, physical examination, and questionnaire data from a total of 39,156 participants in NHANES from 2011 to 2018. Subsequently, individuals less than 18 years old or over 80 years old were excluded, totaling 15,331 individuals. Following this, 1,359 participants lacking information related to coronary artery disease (CAD) were excluded. Next, we removed 13,342 participants due to missing data required for the calculation of the HRR. To further enhance the credibility of the study, we continued to screen for potential confounding factors, leading to the exclusion of an additional 2,243 participants who had missing information regarding these factors. Specifically, six individuals did not provide information on smoking, 219 lacked measured systolic blood pressure, 273 were missing data related to hypertension, 2 lacked glycated hemoglobin data, 4 had unknown levels of education, 106 did not have LDL data, 895 were missing Poverty Income Ratio (PIR) income level data, 2 lacked diagnosed diabetes-related information, and another 732 did not provide information on alcohol consumption. Ultimately, a total of 6,881 participants were included in the study.

### Definition of CAD

The diagnosis of CAD was derived from the questionnaire data in the NHANES database. It was primarily determined by three questions in the Medical Conditions Section: 1) Have you ever been told you had coronary heart disease? 2) Have you ever been told you had angina or angina pectoris? 3) Have you ever been told you had a heart attack? This is also called a myocardial infarction. An affirmative response to any of the above three questions was used to define CAD, and the absence of such a response was considered negative for CAD.

### Definition of HRR

The Hemoglobin-to-Red Blood Cell Distribution Width Ratio (HRR) is calculated by dividing the Hemoglobin (HB) levels by the Red Blood Cell Distribution Width (RDW), utilizing laboratory data from NHANES. In subsequent analyses, HRR is stratified into quartiles.

### Covariates

Building on previous relevant studies, this study included hypertension, waist circumference (WC), systolic blood pressure (SBP), fasting plasma glucose (FPG), triglycerides (TG), low-density lipoprotein (LDL), marital status, body mass index (BMI), total cholesterol (TC), age, poverty-income ratio (PIR), glycated hemoglobin (HbA1C), diabetes, race, education level, high-density lipoprotein (HDL), and gender as covariates.

BMI is calculated from anthropometric data, specifically height (HT in meters, m) and weight (WT in kilograms, kg), using the formula BMI = WT/(HT)^2. Marital status is categorized into three main groups: 1) Married/cohabiting, 2) Widow/widower/separated, and 3) Unmarried. Race is classified into five categories based on NHANES survey results: Mexican, Hispanic, White, Black, and Other Race. Education level is primarily divided into three categories: Less than high school, High school, and More than high school.

Hypertension is defined by three main criteria: 1) Systolic blood pressure of 140 mmHg or higher in anthropometric data, 2) Diastolic blood pressure of 90 mmHg or higher in anthropometric data, and 3) Participants who have been told by a doctor that they have hypertension, as reported in the blood pressure questionnaire. Meeting any one of these criteria is sufficient to be defined as having hypertension.

Diabetes is defined by four criteria: 1) FPG of 7 mmol/L or higher, 2) HbA1C of 6.5% or higher, 3) Participants who have been told by a doctor that they have diabetes, as reported in the questionnaire, and 4) Participants who have taken hypoglycemic medications, as reported in the questionnaire. Meeting any one of these criteria is sufficient to be defined as having diabetes.

Gender and age are well-known demographic factors that influence the prevalence and outcomes of coronary artery disease (CAD). The Poverty Income Ratio (PIR) and level of education reflect socioeconomic status, which is associated with health outcomes, including cardiovascular diseases. Race is a significant factor affecting health disparities and the prevalence of CAD. Alcohol use and smoking status indicate that both drinking and smoking are risk factors for CAD. Body Mass Index (BMI) shows that obesity is a known risk factor for CAD. Lipids such as high-density lipoprotein, triglycerides, and low-density lipoprotein are directly related to cardiovascular health. Systolic blood pressure (SBP) indicates that hypertension is a major risk factor for CAD. HbA1c and fasting plasma glucose (FPG) suggest that glycemic control is crucial in CAD, especially among diabetic patients. Waist circumference (WC), as a measure of central obesity, is associated with an increased risk of CAD. Marital status has been shown to affect health behaviors and outcomes, including CAD. Lastly, hypertension and diabetes, as well-recognized comorbidities, significantly increase the risk of CAD.

### Statistical analysis

In this study, we conducted thorough processing and quality control of the data. Given the non-normal distribution of the data, continuous variables are expressed as medians with interquartile ranges (Q1, Q3), while categorical variables are presented as counts and percentages. The Kruskal-Wallis test was used to compare continuous variables between the CAD and non-CAD groups, and the chi-squared test was used for categorical variables. A p-value less than 0.05 was considered to indicate a statistically significant difference.

Using logistic regression analysis appropriate for the survey design, we explored the relationship between HRR and CAD. The robustness of the model was incrementally enhanced by adjusting for different covariates. Specifically, Model 1 is the most basic model, with no adjustments for any variables. Model 2 is adjusted for race, age, and gender. Finally, Model 3 includes adjustments for potential confounding factors such as BMI, HDL, gender, HbA1C, WC, age, PIR, TG, race, LDL, education level, TC, FPG, SBP, hypertension, and diabetes to further strengthen the robustness of the model.

## Results

### Demographic and initial characteristics of participants

The study selected 6,881 adult Americans from the NHANES database spanning from 2011 to 2018, with a higher number of males, totaling 3,449 individuals, accounting for approximately 50.12% of the study population. Within this cohort, 499 individuals were diagnosed with CAD, representing 7.25% of the sample size. The majority of participants were identified as non-Hispanic white people, with 2,807 individuals making up 40.79% of the total study cohort. Table 1 summarizes the demographic and baseline characteristics of the study subjects, stratified by HRR quartiles (Table 1). Individuals with higher HRR tended to be younger (*P* < 0.001), have a smaller waist circumference (*P* < 0.001), higher income (*P* < 0.001), lower BMI (*P* < 0.001), drink less alcohol (*P* < 0.001), have a higher level of education (*P* = 0.007), and were free from hypertension (*P* < 0.001). Additionally, individuals with higher HRR levels may have lower HDL levels (P < 0.001), higher LDL levels (P < 0.001), and higher TG levels (P < 0.01) in their blood.

**TABLE 1:** Characteristics of the participants categorized by HRR.

HRR	Q1 (0.27–0.96)	Q2 (0.96–1.06)	Q3 (1.06–1.16)	Q4 (1.16–1.50)	P-value
WC, cm	100.00 (65.50–176.00)	98.10 (63.10–169.50)	96.50 (64.00–161.60)	98.20 (64.20–172.50)	<0.001
HDL, mmol/L	1.40 (0.54–5.84)	1.40 (0.16–3.93)	1.32 (0.44–3.21)	1.24 (0.41–4.47)	<0.001
TG, mmol/L	0.96 (0.16–4.50)	1.05 (0.11–4.49)	1.09 (0.23–4.52)	1.23 (0.24–4.49)	<0.001
LDL, mmol/L	2.66 (0.39–9.70)	2.77 (0.65–7.45)	2.87 (0.46–6.21)	2.94 (0.36–9.23)	<0.001
Age, years	54.00 (20.00–80.00)	54.00 (20.00–80.00)	49.00 (20.00–80.00)	43.00 (20.00–80.00)	<0.001
PIR	1.84 (0.02–5.00)	2.24 (0.01–5.00)	2.27 (0.01–5.00)	2.30 (0.01–5.00)	<0.001
BMI, kg/m2	29.40 (15.50–64.50)	28.00 (15.40–68.70)	27.30 (15.70–70.10)	27.70 (16.20–67.50)	<0.001
SBP, mmHg	124.00 (66.00–238.00)	122.00 (84.00–224.00)	120.00 (74.00–198.00)	120.00 (86.00–236.00)	<0.001
HBA1C, %	5.70 (3.60–17.00)	5.60 (4.10–15.20)	5.50 (4.20–16.50)	5.40 (3.70–16.50)	<0.001
FPG, mmol/L	5.61 (1.17–23.40)	5.55 (3.16–23.80)	5.61 (3.00–25.00)	5.66 (2.17–25.20)	0.020
RDW, %	14.60 (12.00–29.20)	13.50 (11.10–17.90)	13.10 (11.30–15.60)	12.70 (11.00–16.00)	<0.001
HB, g/dL	12.50 (6.10–16.00)	13.70 (11.20–17.90)	14.60 (12.40–17.80)	15.70 (12.90–19.60)	<0.001
HRR	0.88 (0.27–0.96)	1.02 (0.96–1.06)	1.11 (1.06–1.16)	1.22 (1.16–1.50)	<0.001
Race, n (%)					<0.001
Mexican	189 (11.01)	228 (13.25)	236 (13.76)	257 (14.92)	
Hispanic	172 (10.02)	213 (12.38)	194 (11.31)	151 (8.77)	
White	500 (29.14)	641 (37.25)	808 (47.11)	858 (49.83)	
Black	657 (38.29)	390 (22.66)	231 (13.47)	141 (8.19)	
Other Race	198 (11.54)	249 (14.47)	246 (14.34)	315 (18.29)	
Education, n (%)					0.007
Under high school	388 (22.61)	365 (21.21)	324 (18.89)	320 (18.58)	
High school	394 (22.96)	348 (20.22)	374 (21.81)	394 (22.88)	
Over high school	934 (54.43)	1008 (58.57)	1017 (59.30)	1008 (58.54)	
Smoking status, n (%)					<0.001
No	1018 (59.32)	978 (56.83)	936 (54.58)	844 (49.01)	
Yes	698 (40.68)	743 (43.17)	779 (45.42)	878 (50.99)	
Alcohol user, n (%)					<0.001
No	265 (15.08)	321 (18.28)	475 (27.05)	611 (34.78)	
Yes	1492 (84.92)	1435 (81.72)	1281 (72.95)	1146 (65.22)	
Gender, n (%)					<0.001
Male	426 (24.83)	580 (33.70)	985 (57.43)	1458 (84.67)	
Female	1290 (75.17)	1141 (66.30)	730 (42.57)	264 (15.33)	
Marital status, n (%)					
Married/cohabiting	905 (52.74)	1054 (61.24)	1096 (63.91)	1099 (63.82)	
Widow/widower/separated	483 (28.15)	391 (22.72)	307 (17.90)	255 (14.81)	
Unmarried	328 (19.11)	276 (16.04)	312 (18.19)	368 (21.37)	
Hypertension, n (%)					<0.001
No	806 (46.97)	971 (56.42)	1098 (64.02)	1150 (66.78)	
Yes	910 (53.03)	750 (43.58)	617 (35.98)	572 (33.22)	
Diabetes, n (%)					<0.001
No	1256 (73.19)	1376 (79.95)	1432 (83.50)	1476 (85.71)	
Yes	460 (26.81)	345 (20.05)	283 (16.50)	246 (14.29)	
CAD, n (%)					<0.001
No	1536 (89.51)	1572 (91.34)	1619 (94.40)	1648 (95.70)	
Yes	180 (10.49)	149 (8.66)	96 (5.60)	74 (4.30)	

Data were expressed as n (%) and median (interquartile range).

HRR, hemoglobin-to-red blood cell distribution width ratio, WC, waist circumference; HDL, high-density lipoprotein; TG, triglycerides; LDL, low density lipoprotein; PIR, poverty income ratio; BMI, body mass index; SBP, systolic blood pressure; CAD coronary artery disease.

### Univariate logistic analysis reveals associations between multiple variables and CAD

As shown in Table 2, in the univariate analysis, WC (OR = 1.02, 95%CI: 1.02, 1.03, *P* < 0.0001), TG (OR = 1.28, 95%CI: 1.15, 1.43, *P* < 0.0001), SBP (OR = 1.02, 95%CI: 1.02, 1.03, *P* < 0.0001), FPG (OR = 1.16, 95%CI: 1.13, 1.20, *P* < 0.0001), BMI (OR = 1.02, 95%CI: 1.02, 1.03, *P* = 0.0004), RDW (OR = 1.27, 95%CI: 1.20, 1.33, *P* < 0.0001), HRR (OR = 1.13, 95%CI: 1.09, 1.16, *P* < 0.0001) were positively associated with CAD. In contrast, LDL (OR = 0.54, 95%CI: 0.49, 0.61, *P* < 0.0001) and HB (OR = 0.92, 95%CI: 0.86, 0.97, *P* = 0.0033) were negatively associated with CAD (Table 2).

**TABLE 2:** Weighted univariate logistic analyses between Variables and CAD (odds ratios, 95% confidence intervals).

Variables	Univariate analysis (crude model)	
	OR95% CI	P-value
WC	1.02 (1.02, 1.03)	<0.0001
BMI	1.02 (1.01, 1.03)	0.0004
TG	1.28 (1.15, 1.43)	<0.0001
LDL	0.54 (0.48, 0.61)	<0.0001
SBP	1.02 (1.02, 1.03)	<0.0001
FPG	1.16 (1.13, 1.20)	<0.0001
RDW	1.27 (1.20, 1.33)	<0.0001
HB	0.92 (0.86, 0.97)	0.0033
HRR	1.13 (1.09, 1.16)	<0.0001

### The relationship between CAD and HRR quartiles

Referencing Table 3, elevated HRR quartiles are associated with a decreased risk of CAD (Table 3). The unadjusted model reveals an inverse relationship between HRR quartiles and the prevalence of CAD (Model 1). This correlation remains significant after partial adjustments in Model 2. With comprehensive consideration of all potential confounding factors, the OR for CAD in relation to the second (Q2), third (Q3), and fourth (Q4) quartiles compared to the reference quartile (Q1) are approximately 0.38 (95% CI: 0.29–0.51), 0.42 (95% CI: 0.30–0.58), and 0.51 (95% CI: 0.38–0.70), respectively, for Model 3 (*P* < 0.001). Additionally, under strict control for potential confounding variables, at the HRR Q4 level, each unit increase in HRR corresponds to a 49% reduction in the probability of CAD.

**TABLE 3:** Multivariate Cox regression analysis of HRR with CAD.

	HRR quartiles				P for trend
	Q1	Q2	Q3	Q4	
	0.27–0.96	0.96–1.06	1.06–1.16	1.16–1.50	
	OR (95%CI)	OR (95%CI)	OR (95%CI)	OR (95%CI)	
CAD	180 (10.49%)	149 (8.66%)	96 (5.60%)	74 (4.30%)	
Model 1	Reference	0.76 (0.64, 0.90)	0.51 (0.39, 0.65)	0.38 (0.29, 0.51)	<0.001
Model 2	Reference	0.75 (0.66, 0.86)	0.50 (0.38, 0.67)	0.42 (0.30, 0.58)	<0.001
Model 3	Reference	0.71 (0.58, 0.88)	0.64 (0.48, 0.86)	0.51 (0.38, 0.70)	<0.001

Values are presented as weighted odds ratios (ORs), 95% confidence interval (95%CI), and *P* value.

Model 1 adjusted for none.

Model 2 adjusted for gender, age and race.

Model 3 adjusted for gender, age, PIR, education level, race, alcohol user, smoking status, BMI, HDL, TG, LDL, SBP, HBA1C, FPG, WC, marital status, hypertension and diabetes.

**FIGURE 2:**
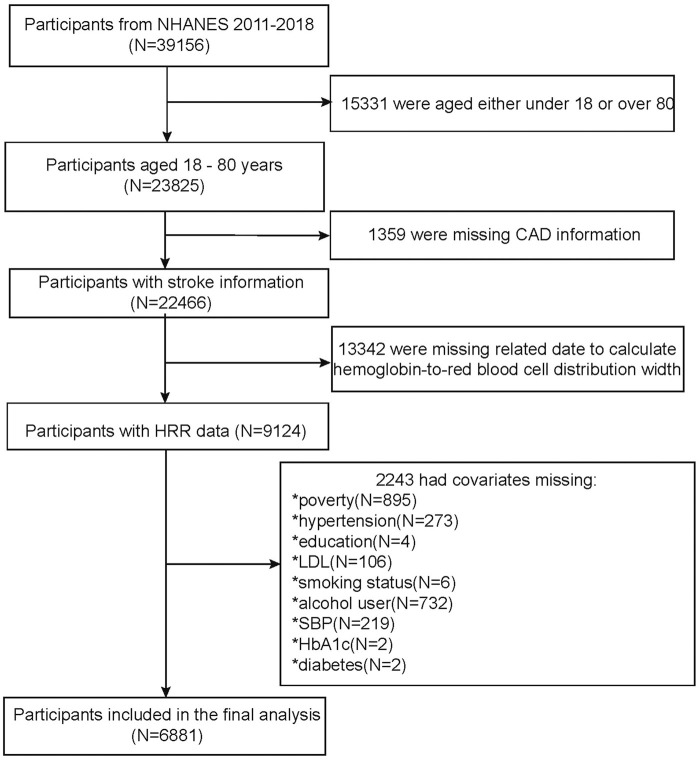
Relationship between HRR and CAD.

Using smooth curve fitting and threshold effect analysis, we assessed the potential linear correlation between HRR and the risk of CAD. As shown in Figure 2, a significant linear relationship was found between these variables (Figure 2). Table 4 details the outcomes from a standard linear regression model, revealing a 74% reduction in CAD risk for each one-unit increment in HRR (*P* = 0.0002). Two-piecewise linear regression models identified a critical point at an HRR value of 1.02. For HRR values exceeding 1.02, each unit increase in HRR is associated with a 91% decrease in CAD odds (OR = 0.09, 95%CI = 0.02–0.43, *P* = 0.0026), while for HRR values at or below 1.02, each unit increase in HRR is associated with a 53% decrease in CAD odds (OR = 0.47, 95%CI = 0.17–1.35, *P* = 0.1626). The log-likelihood ratio test yielded a P value of 0.131. Consequently, when synthesizing these findings, a definite inverse linear relationship is observed between HRR and CAD risk (Table 5). This implies that for individuals with an HRR value exceeding 1.02, increasing body water content and reducing blood viscosity to a certain extent may potentially lower the risk of CAD. However, such measures may not be effective in individuals with an HRR equal to or below 1.02.

**TABLE 4:** Threshold and saturation effect analysis of HRR on CAD.

Outcomes	CAD	
	OR (95%CIs)	P value
Model a		
(Fitting model by standard linear regression)	0.26 (0.13, 0.53)	0.0002
Model b		
(Fitting models by two-piecewise linear regression)		
Infection point	1.02	
<Infection point	0.47 (0.17, 1.35)	0.1626
>Infection point	0.09 (0.02, 0.43)	0.0026
P for log-likelihood ratio test	0.131	

The two-piecewise regression models were adjusted for gender, age, PIR, education level, race, alcohol user, smoking status, BMI, HDL, TG, LDL, SBP, HBA1C, FPG, WC, marital status, hypertension and diabetes.

OR, odds ratios, 95% CI, 95% confidence interval.

**TABLE 5:** Stratified analysis of the correlation between HRR and CAD in adults in the NHANES 2013–2018.

Subgroup	CAD	
	OR (95%CI)	P-value
Gender, n (%)		
Male	0.01 (0.01, 0.03)	<0.0001
Female	0.29 (0.12, 0.73)	0.0084
Marital status		
Married/cohabiting	0.15 (0.07, 0.30)	<0.0001
Widow/widower/separated	0.20 (0.08, 0.49)	0.0004
Unmarried	0.11 (0.02, 0.82)	0.0310
Education		
Under high school	0.05 (0.02, 0.14)	<0.0001
High school	0.25 (0.09, 0.69)	0.0077
Over high school	0.21 (0.10, 0.47)	0.0001
Alcohol user		
No	0.54 (0.19, 1.55)	0.2505
Yes	0.09 (0.05, 0.17)	<0.0001
Smoking status		
No	0.23 (0.10, 0.54)	0.0007
Yes	0.07 (0.04, 0.15)	<0.0001
Hypertension		
No	0.41 (0.12, 1.36)	0.1453
Yes	0.20 (0.11, 0.38)	<0.0001
Diabetes, n (%)		
No	0.14 (0.07, 0.27)	<0.0001
Yes	0.31 (0.13, 0.74)	0.0080

## Discussion

CAD is a prevalent condition in cardiovascular medicine, encompassing a spectrum of pathologies from coronary atherosclerosis to angina and myocardial infarction (Liu et al., 2023; Larsen et al., 2015). According to the World Health Organization, CAD accounts for 7.4 million deaths globally, highlighting its significant impact on individual health and socio-economics (Dromain et al., 2013). Among the multifaceted etiologies of CAD, inflammation is a pivotal factor (Kryczka et al., 2021; Sun et al., 2024). Our study, leveraging data from NHANES between 2011 and 2018, reveals a significant negative correlation between the HRR and the likelihood of CAD, indicating an approximate linear association.

Inflammation is a cornerstone in the pathophysiology of CAD, a condition inherently linked with inflammatory processes (Gadidala et al., 2023). Both HRR and RDW, recognized as novel inflammatory biomarkers, have been implicated in inflammation and are associated with the incidence and adverse outcomes of various diseases, including CAD (Ackland et al., 2018). Previous research has demonstrated the utility of RDW in predicting mortality rates among CAD patients. A study examining 1,039 outpatients from 1997 to 2007, with follow-up through 2009, revealed that patients in the highest RDW quartile had a 66% higher risk of death compared to those in the lowest quartile, with a 1% increase in RDW associated with a 10% increase in all-cause mortality risk (Qiu et al., 2017). RDW emerged as an independent prognostic indicator for CAD patients (Fava et al., 2019) A study on non-small cell lung cancer (NSCLC) included 245 NSCLC patients, 97 patients with benign pulmonary nodules, and 94 healthy volunteers, analyzing the relationship between various hematological indicators such as red blood cell distribution width (RDW) and tumor progression. It was found that RDW could be used to distinguish NSCLC patients from healthy controls (p < 0.05). RDW was positively correlated with NSCLC staging, while the hemoglobin-to-red blood cell distribution width ratio (HRR) was negatively correlated with NSCLC staging. The study concluded that RDW could serve as a simple and effective biomarker for diagnosing and assessing the progression of NSCLC (Chen et al., 2020). A study has investigated the association between dietary patterns of patients with coronary artery disease and their sociodemographic and health-related characteristics. The study included 250 coronary artery disease patients who were aged 40 and above. It was found that dietary patterns are related to sociodemographic and health-related factors (Esmaili et al., 2015). Another study conveniently recruited 124 full-time male and female firefighters from the Fire and Rescue Services in Cape Town, South Africa, between September and November 2020. It was found that alcohol consumption was significantly associated with gender, race, total minutes of low-intensity physical activity, diastolic blood pressure, and hypertension (p = 0.005). Gender (p = 0.021) and race (p = 0.042) were significantly related to the type of alcohol consumed. Alcohol intake was a significant predictor of total low-intensity physical activity as well as systolic blood pressure (p = 0.048) and diastolic blood pressure (p = 0.036). This suggests that targeting physical activity may potentially improve Coronary Artery Disease (CAD) (Dąbek et al., 2020).

The role of HRR in CAD, however, has been less clear. Our study, being the first to explore the potential link between HRR and CAD using a large dataset from NHANES, found that high levels of HRR are protective against CAD, reducing the probability of CAD by 56% for each unit increase in HRR. This contrasts with findings in other disease contexts, such as nasopharyngeal carcinoma, where HRR was associated with poorer outcomes. The discrepancy may be attributed to the different physiological responses to disease stimuli in oncological versus cardiovascular settings, suggesting that the role of HRR may vary significantly across disease types.

Under stringent inclusion criteria, our study meticulously selected indicators and populations from the reputable NHANES database, ensuring a certain level of persuasiveness in our results. We endeavored to adjust for a range of covariates to ensure the reliability of our findings. However, it is important to acknowledge the limitations inherent in our study. The cross-sectional nature of the study precludes the establishment of causal relationships between HRR and CAD, limiting our exploration to potential associations. HRR is primarily determined by two factors, HB and RDW, with HB being a relatively easily modifiable indicator. In the elderly, whose blood is relatively viscous, moderately diluting blood viscosity and increasing body water content within the normal range of HB could potentially reduce HRR to some extent, thereby reducing the risk of CAD. Of course, these hypotheses require further validation through multicenter clinical trials. Additionally, while we controlled for multiple covariates, there may be other confounding factors that could influence the relationship between HRR and CAD, which were not accounted for in this study. Despite these limitations, our study is novel in identifying HRR as a potential protective factor against CAD, providing a foundation and new insights for future research.

## Conclusion

In conclusion, this study demonstrates a significant inverse linear relationship between the HRR and the risk of CAD, suggesting that higher HRR values are associated with a reduced likelihood of CAD. These findings provide valuable insights into the role of inflammation in CAD and highlight HRR as a potential inflammatory marker that could aid in CAD risk stratification.

## Data Availability

Publicly available datasets were analyzed in this study. This data can be found here: The data analyzed in this study are publicly available from the National Health and Nutrition Examination Survey (NHANES), conducted by the Centers for Disease Control and Prevention (CDC). You can access the datasets through the NHANES website: Repository: NHANES Data and Documentation Access Link: https://wwwn.cdc.gov/nchs/nhanes/.
