# Correction: Hemoglobin-to-red blood cell distribution width ratio as a protective factor against coronary artery disease: a cross-sectional analysis of NHANES (2011–2018)

**DOI:** 10.3389/fphar.2025.1634607

**Published:** 2025-06-10

**Authors:** Xin-Da Wang, Chaoya Li, Jia Hu, Fen Cao, Li Zhu, Yongzhi Zhu, Zhongzheng Wen, Jun Liu

**Affiliations:** ^1^ Department of Cardiology, Chenzhou First People’s Hospital, Chenzhou, China; ^2^ Hunan University of Medicine General Hospital, Huaihua, China; ^3^ Department of Cardiology, Chengdu First People’s Hospital, Chengdu, China

**Keywords:** coronary artery disease, hemoglobin-to-red blood cell distribution width ratio, inflammation, NHANES, cross-sectional study

In the published article, there was an error in **Affiliation 1**. Instead of “Department of Cardiology, Chengdu First People’s Hospital, Chengdu, Sichuan, China”, it should be “Department of Cardiology, Chenzhou First People’s Hospital, Chenzhou, China”.

In the published article, there was an error regarding the **Affiliation** for Zhongzheng Wen. Instead of having affiliation 1, they should have 3 Department of Cardiology, Chengdu First People’s Hospital, Chengdu, China.

In the published article, the **Equal Contribution** note “† These authors contributed equally to this work” was omitted for authors Xin-Da Wang, Chaoya Li and Jia Hu. The corresponding symbol † has been added to the author list.

The authors apologize for these errors and state that these do not change the scientific conclusions of the article in any way. The original article has been updated.

